# Executive Functions in Latin American Older Adults: Exploring the Concept Shifting Test and Its Relationship with the *APOE Gene* in a Cross‐Sectional Sample from the LatAm‐FINGERS Study

**DOI:** 10.1002/alz70857_105158

**Published:** 2025-12-26

**Authors:** Davi Melillo Cardoso, Clarisse Vasconcelos Friedlaender, Monica Sanches Yassuda, Leonardo Cruz de Souza, Luc¡a Crivelli, Claudia Kimie Suemoto, Maria Eugenia Martin, Ismael Luis Calandri, Natalia Acosta‐Baena, Ezequiel Ignacio Surace, Andrés Damian, Maria Da Graça Morais Martin, Ana Vigil‐Martinez, Gustavo Henrique Cançado, Rosa Maria Salinas‐Contreras, Belen Custodio, María Isabel Cusicanqui, Carolina Delgado, Jorge M Leon‐Salas, Lissette Duque, Nilton Custodio, Ivonne Z. Jimenez Velazquez, Ana Charamelo, Daisy Miguelina Acosta, David Fernando Aguillón Niño, Gustavo Sevlever, Ricardo Allegri, Ricardo Nitrini, Ana Luisa Sosa, Karina Braga Gomes, Andreia de Andrades, Dias Teixeira, Lais Soares Figueiredo, Letícia Maria Ferreira de Souza, Vinicius Ribeiro Jeunon, Maira Tonidandel Barbosa, Vitoria Silva Vieira, Andre Luis Lopes, João Victor de Faria Rocha, Marcelo Machado Prates, Jessica Tossati, Sonia Maria Dozzi Brucki, Heather M Snyder, Maria C. Carrillo, Miia Kivipelto, Paulo Caramelli

**Affiliations:** ^1^ Faculdade de Medicina, Universidade Federal de Minas Gerais, Belo Horizonte, Brazil, Belo Horizonte, Minas Gerais, Brazil; ^2^ Universidade Federal de Minas Gerais, Belo Horizonte, Minas Gerais, Brazil; ^3^ University of São Paulo, São Paulo, Brazil; ^4^ Federal University of Minas Gerais, Belo Horizonte, Minas Gerais, Brazil; ^5^ Institute of Neurosciences (INEU), Fleni‐CONICET, Buenos Aires, Buenos Aires, Argentina; ^6^ Division of Geriatrics, Department of Internal Medicine, University of São Paulo Medical School, São Paulo, São Paulo, Brazil; ^7^ FLENI, Buenos Aires, Argentina; ^8^ Fleni, Buenos Aires, Buenos Aires, Argentina; ^9^ Grupo de Neurociencias de Antioquia, Universidad de Antioquia, Medellin, Colombia; ^10^ Centro Uruguayo de Imagenología Molecular, CUDIM., Montevideo, Montevideo, Uruguay; ^11^ LIM44, Hospital das Clinicas HCFMUSP, Faculdade de Medicina, Universidade de Sao Paulo, Sao Paulo, Sao Paulo, Brazil; ^12^ National Insititute of Medical Sciences and Nutrition, Mexico City, DF, Mexico; ^13^ Laboratorio de demencias del Instituto Nacional de Neurología y Neurocirugía Manuel Velasco Suárez, México, Mexico city, DF, Mexico; ^14^ Escola de Educação Física, Fisioterapia e Terapia Ocupacional, Universidade Federal de Minas Gerais, Belo Horizonte, Brazil, Belo Horizonte, Minas Gerais, Brazil; ^15^ Laboratorio de demencias del Instituto Nacional de Neurología y Neurocirugía Manuel Velasco Suárez, Ciudad de Mexico, Mexico; ^16^ Unidad de Investigación de Deterioro Cognitivo y Prevención de Demencia, Instituto Peruano de Neurociencias, Lima, Lima, Peru; ^17^ Centro Neurológico Mente Activa, La Paz, Bolivia (Plurinational State of); ^18^ Hospital Clínico Universidad de Chile, Departamento de Neurología y Neurocirugía, Santiago, Santiago, Chile; ^19^ Clínica Bíblica Hospital, San José, Costa Rica; ^20^ Global Brain Health Institute, Trinity College Dublin, dublin, Ireland; ^21^ Neuromedicenter, Quito, Ecuador; ^22^ Centro de Investigación en Geriatría, Recinto de Ciencias Médicas, Universidad de Puerto Rico, San Juan, PR, USA; ^23^ Hospital Británico, Clínica de Memoria, Montevideo, Montevideo, Uruguay; ^24^ Departamento de Neuropsicología, Facultad de Medicina‐Hospital de Clínicas, Universidad de la República, Montevideo, Uruguay; ^25^ Universidad Nacional Pedro Henriquez Urena, Santo Domingo, Dominican Republic; ^26^ Grupo de Neurociencias de Antioquia, Facultad de Medicina, Universidad de Antioquia, Medellín, Antioquia, Colombia; ^27^ Fleni, CABA, Buenos Aires, Argentina; ^28^ Fleni, Buenos Aires, Argentina; ^29^ Hospital das Clínicas da Faculdade de Medicina da Universidade de São Paulo, São Paulo, São Paulo, Brazil; ^30^ Instituto Nacional de Neurología y Neurocirugía, Mexico City, DF, Mexico; ^31^ Faculty of Pharmacy ‐ Federal University of Minas Gerais, Belo Horizonte, Minas Gerais, Brazil; ^32^ Faculty of Medicine ‐ Federal University of Minas Gerais, Belo Horizonte, Minas Gerais, Brazil; ^33^ Behavioral and Cognitive Neurology Unit, Faculdade de Medicina, Universidade Federal de Minas Gerais, Belo Horizonte, Brazil, Belo Horizonte, Minas Gerais, Brazil; ^34^ Universidade Federal de Minas Gerais, Belo Horizonte, Minas gerais, Brazil; ^35^ University of São Paulo School of Medicine, São Paulo, São Paulo, Brazil; ^36^ Alzheimer's Association, Chicago, IL, USA; ^37^ Karolinska University Hospital, Theme Inflammation and Aging, Stockholm, Sweden

## Abstract

**Background:**

Latin America (LA) faces high vulnerability to dementia risk factors. Early detection of cognitive decline, particularly in executive functions (EF), is crucial. The Trail Making Test (TMT) has limitations, while the Concept Shifting Test (CST) offers potential advantages but lacks LA adaptation. This study examines CST's correlation with other EF tests and its relationship with APOE‐ε4 in older adults.

**Methods:**

Cross‐sectional analysis of LatAm‐FINGERS baseline data, including TMT, CST, Stroop Test, and fluency tasks. APOE genotyping used PCR‐RFLP analysis. Jamovi software (v2.3) analyzed correlations (*p* < 0.05).

**Results:**

Sample: 1,143 individuals from 11 LA countries. Mean age = 67.4 years (±4.7), education = 13.2 years (±3.5), 73.9% women, 57.3% mixed‐race. 21.4% carried APOE‐ε4 allele. Mean CST shifting score: 16.0 (±19.3). Significant correlations with age (𝜌=0.085; *p* = 0.009) and education (𝜌 = ‐0.201; *p* <0.001). No sex differences (*p* = 0.172). CST shifting score showed moderate correlation with TMT‐flexibility (𝜌=0.372; *p* <0.001), weak correlations with Stroop (𝜌=‐0.175; *p* <0.001), semantic (𝜌=‐0.133; *p* <0.001) and phonemic fluency (𝜌=‐0.184; *p* <0.001). No significant differences in CST shifting (*p* = 0.951) or TMT‐flexibility (*p* = 0.767) between APOE‐ε4 groups.

**Conclusions:**

Weak correlations between CST shifting, Stroop‐flexibility, and fluency tasks suggest they assess different EF aspects. The moderate CST‐TMT correlation may indicate refined cognitive flexibility measurement in CST. While useful for EF assessment in LA, CST doesn't replace TMT. Diverse tools are crucial for early dementia detection. The lack of association between APOE‐ε4 and cognitive scores emphasizes the importance of assessment regardless of genetic risk.

